# Supplementary material for: Human populations in the world’s mountains: Spatio-temporal patterns and potential controls
Source: PLoS One. 2022 Jul 20;17(7):e0271466. doi: 10.1371/journal.pone.0271466 (PMC9299344; doi:10.1371/journal.pone.0271466)
Supplement: S1 File — In addition, input and output data, code, and high-resolution and supplementary figures are available at: doi.org/10.5281/zenodo.6673651. Spatial outputs (.sqlite) can be easily visualized in QGIS (simply drag and drop). (PDF) [file pone.0271466.s001.pdf]

PLOS ONE

Supplementary Information for

Human populations in the world's mountains: spatio-temporal patterns and topo-climatic controls

Thornton, J.M., Snethlage, M.A., Sayre, R., Urbach, D.R., Viviroli, D., Ehrlich, D., Muccione, V., Wester, P., Insarov, G. Adler, C.

James M. Thornton
E-mail: james.thornton@unibe.ch

This PDF file includes:

Figs. S1 to S19
Tables S1 to S8
SI References

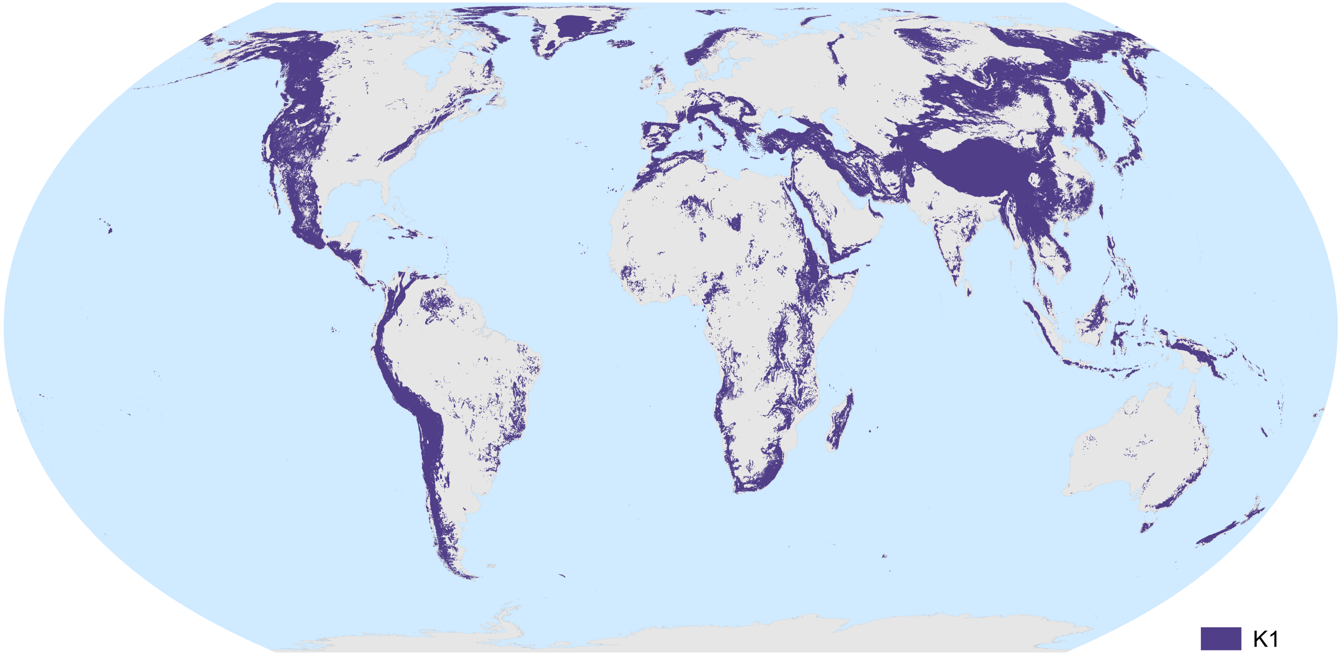

Fig. S1. The global mountain delineation of Kapos et al. (1) ("K1") used in the present study. The Equal Earth coordinate system (EPSG:8857) was used to produce all global maps.

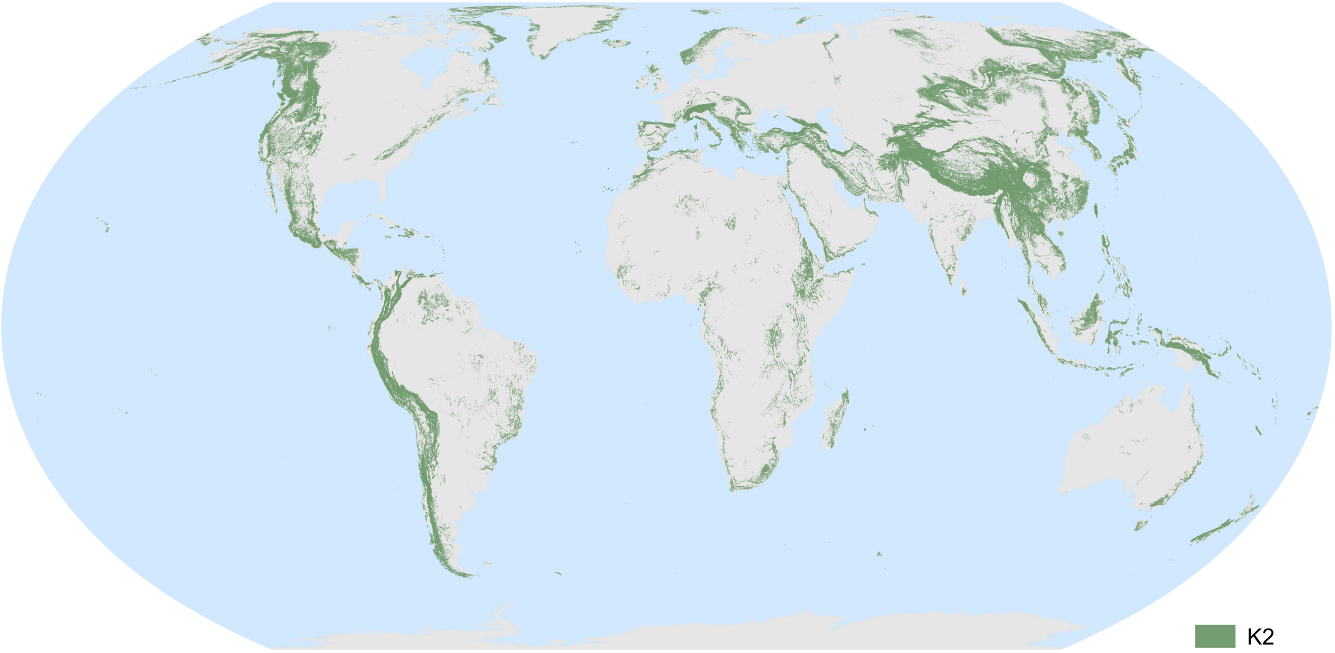

Fig. S2. The new, higher resolution version of a mountain delineation generated using the approach of Körner et al. (2) used in the present study (“K2”). In contrast to the original dataset, no resampling onto a coarser resolution grid was undertaken.

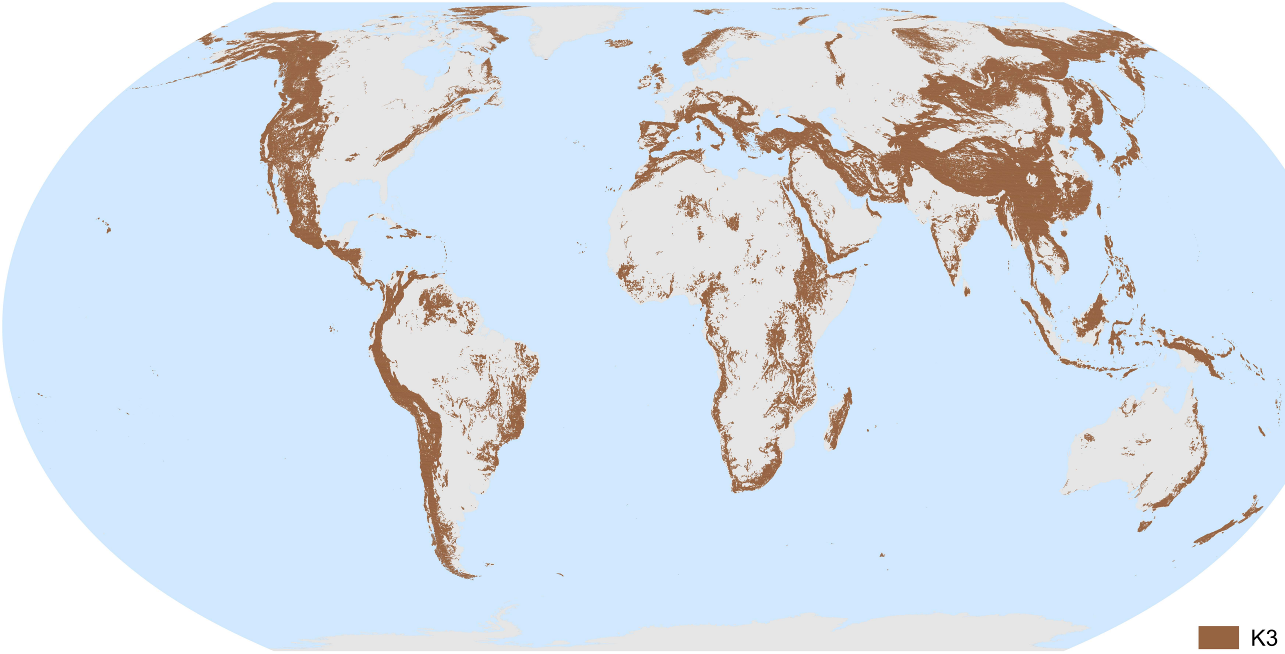

Fig. S3. The global mountain delineation of Karagulle et al. (3) ("K3") that was used in the present study.

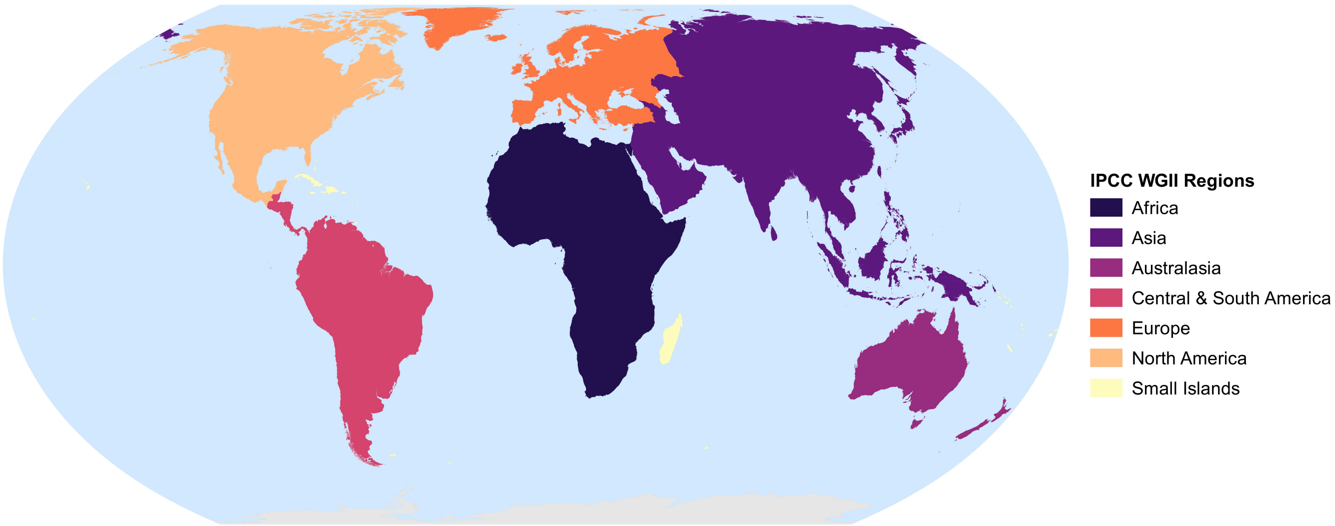

Fig. S4. The seven global regions used by Intergovernmental Panel on Climate Change (IPCC) Working Group II in its 6th Assessment Report. These geometries were one of the datasets used for aggregation and reporting purposes.

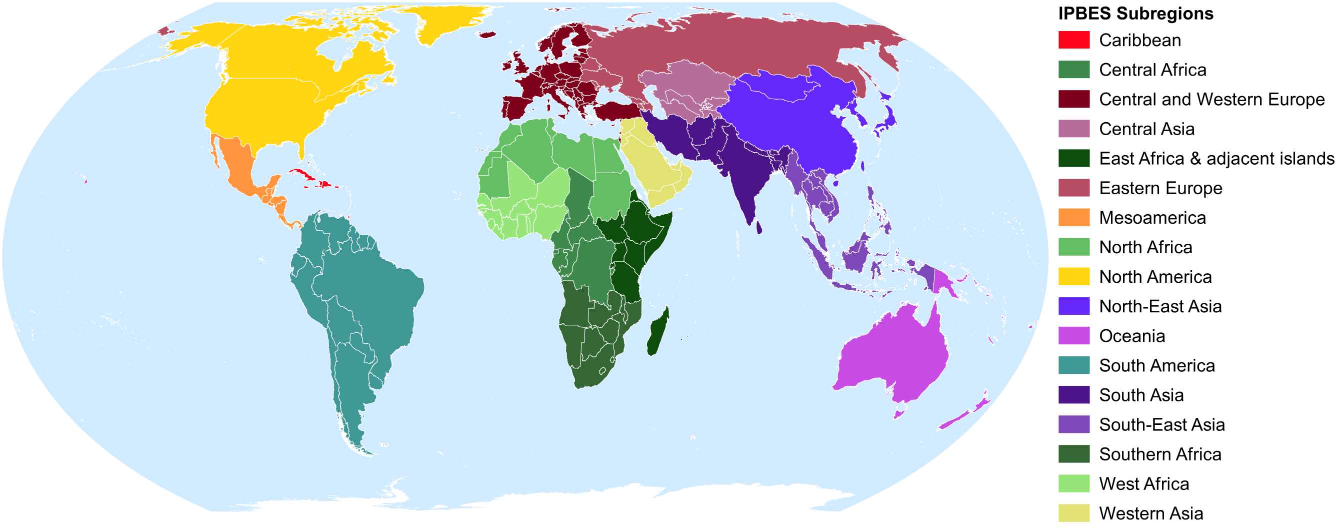

Fig. S5. The 17 sub-regions (solid colors) (exc. Antarctica) and 257 areas (separated by white boundaries) used by the Intergovernmental Science-Policy Platform on Biodiversity and Ecosystem Services (IPBES) to subdivide the global land surface. Source: IPBES. These geometries were one of the datasets used for aggregation and reporting purposes.

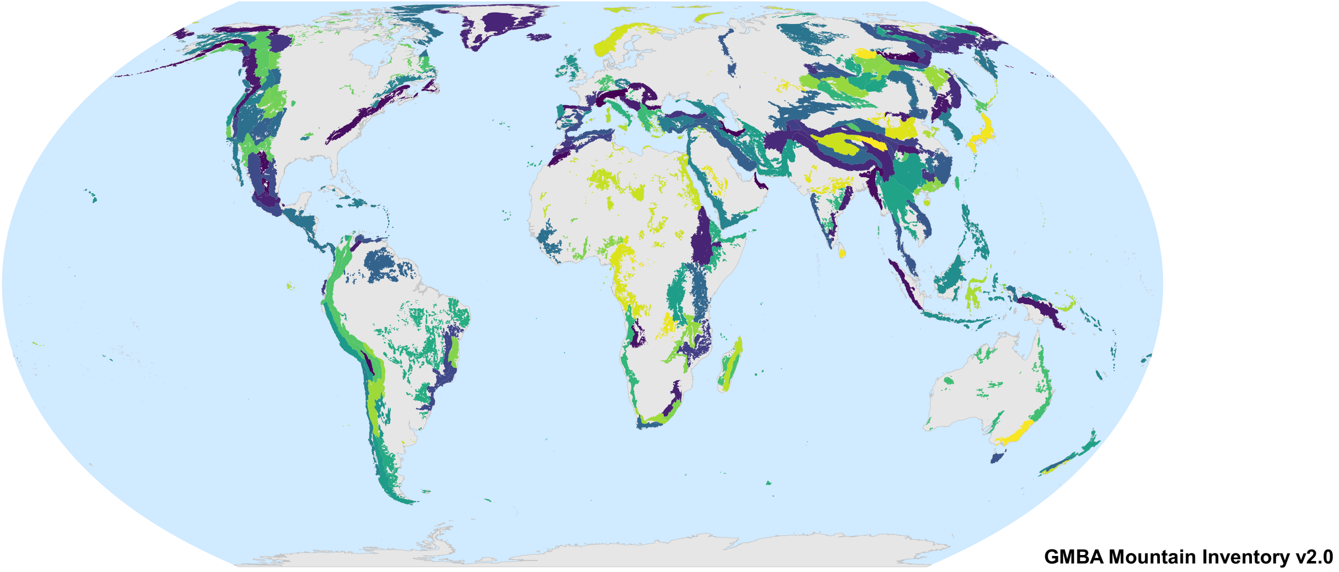

Fig. S6. The 292 polygons of the Global Mountain Biodiversity Assessment's (GMBA's) Mountain Inventory (v2.0). Source: Snethlage et al. (4). Each polygon delineates the extent of a named mountainous region and is placed within a hierarchical structure. These geometries were one of the datasets used for aggregation and reporting purposes.

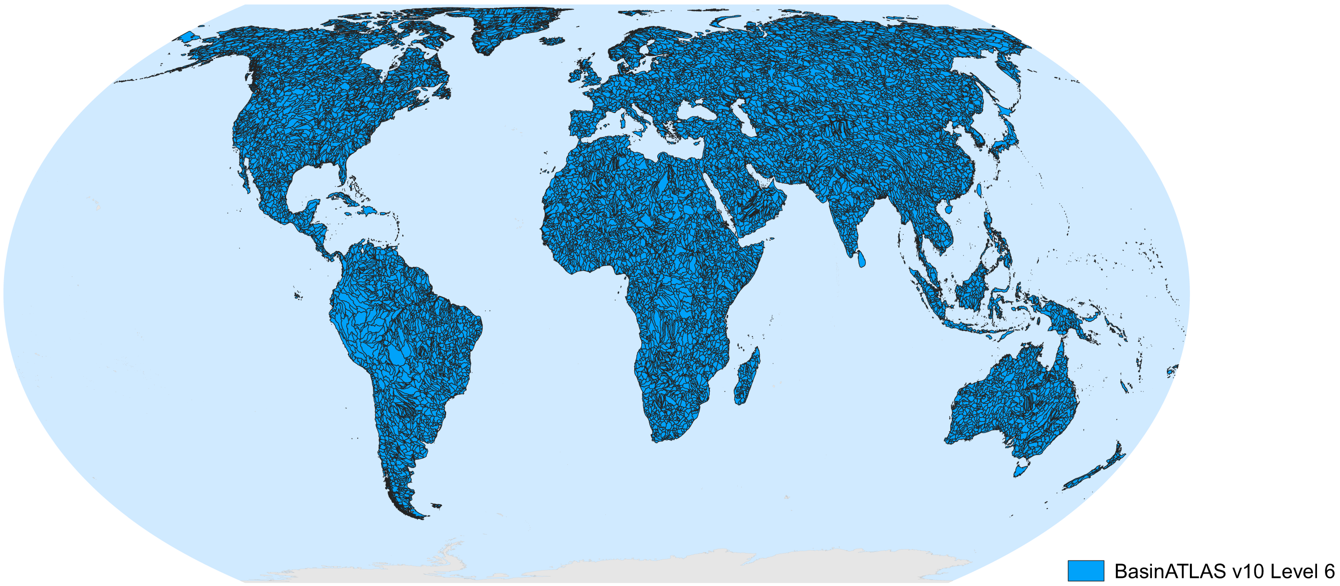

Fig. S7. The 16,397 hydrological basins belonging to the BasinATLAS v10 Level 6 dataset. Source: Linke et al. (5). These geometries were one of the datasets used for aggregation and reporting purposes.

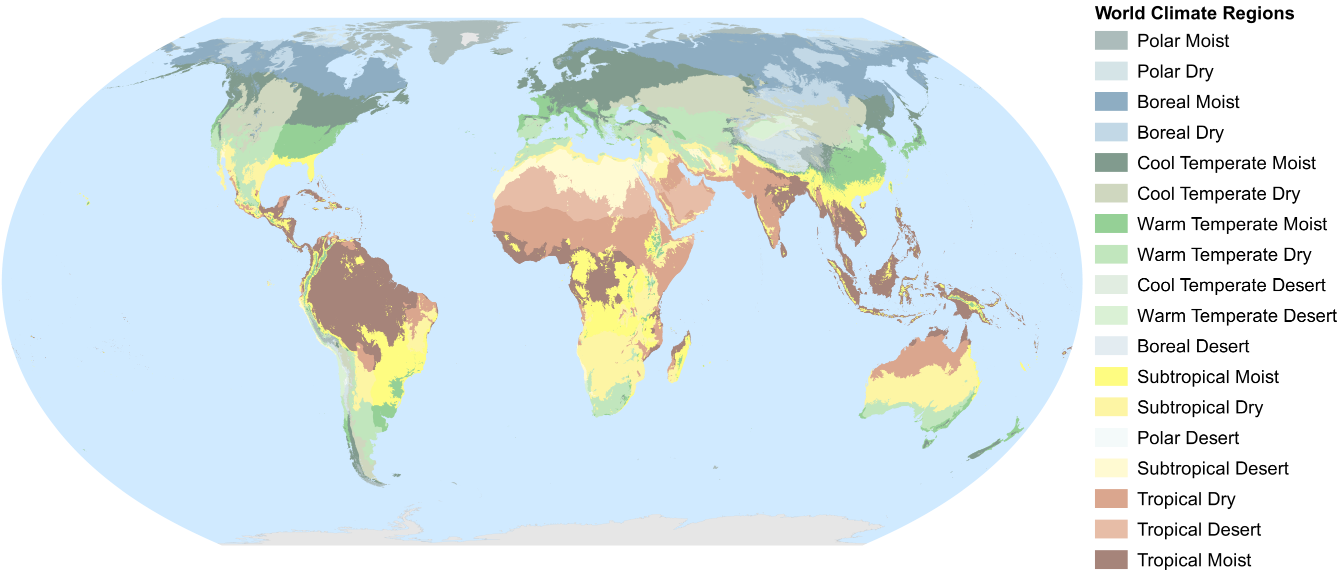

Fig. S8. The spatial distribution of the 18 discrete climatic zones represented in the World Climate Regions dataset. Source: Sayre et al. (6). Note that the "imprint" of mountains is visible in the distribution of the zones. These geometries were one of the datasets used for aggregation and reporting purposes.

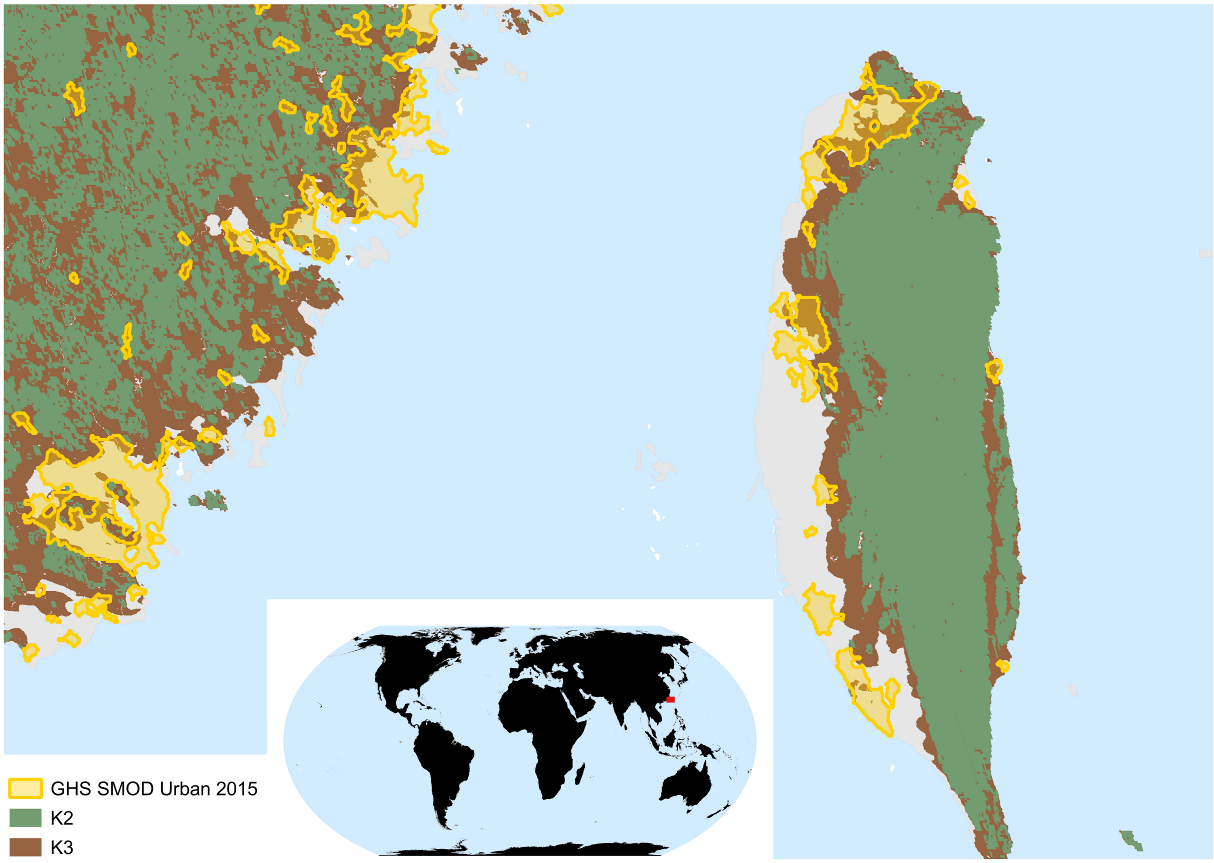

Fig. S9. The extent of the most conservative (K2) and most generous (K3) mountain delineations with respect to 2015 urban extent defined according to the GHS-SMOD dataset. One observes that whilst relatively little of the urban extent fall with K2, considerable areas fall within K3, thus explaining the wide range of urban mountain population reported in Table 1.

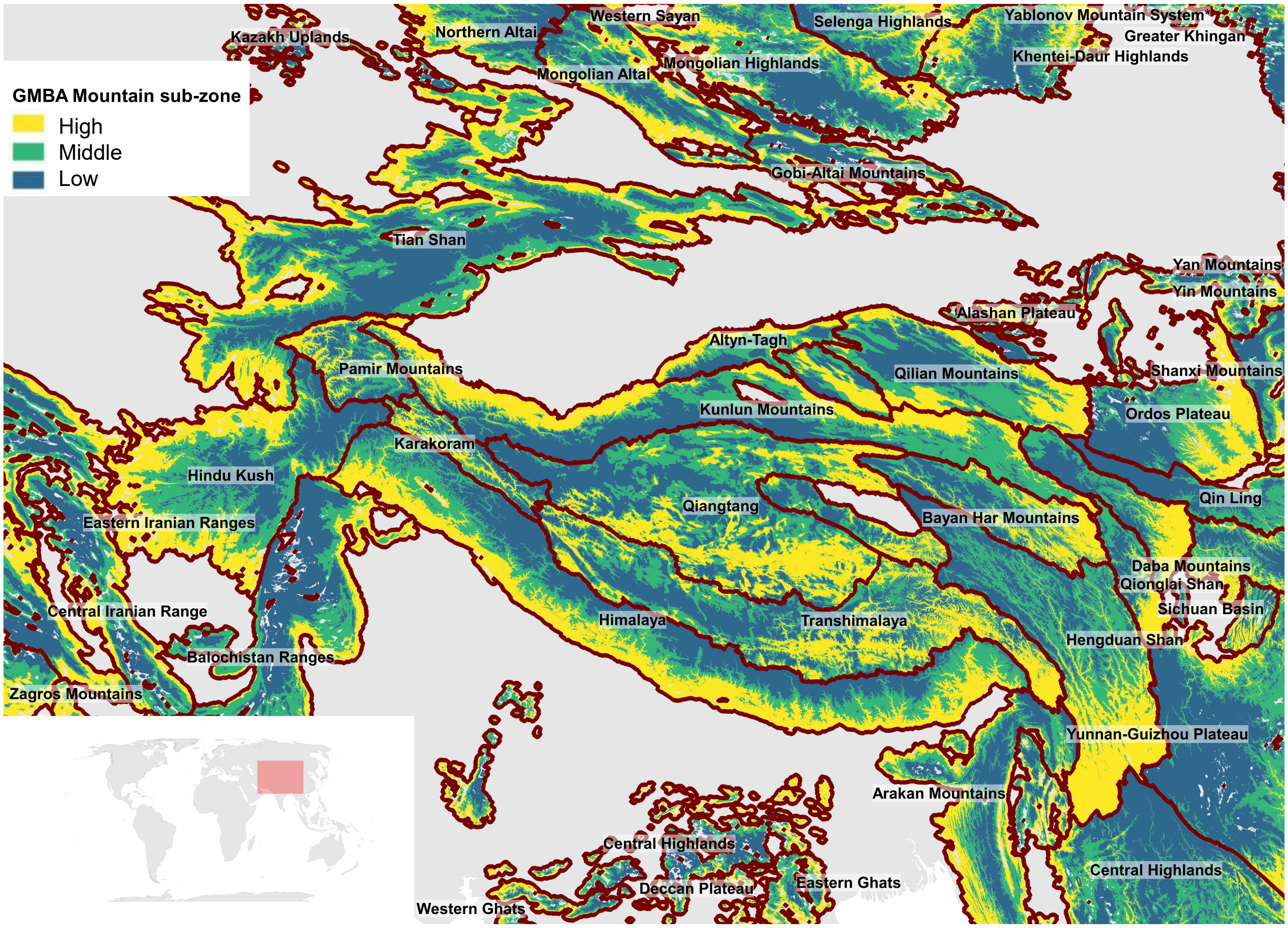

Fig. S10. Illustration of the subdivision of GMBA mountain polygons in the Himalayan region into three sub-zones, based on the quantiles of the elevation ranges within each polygon, for the more spatially granular components of the analysis.

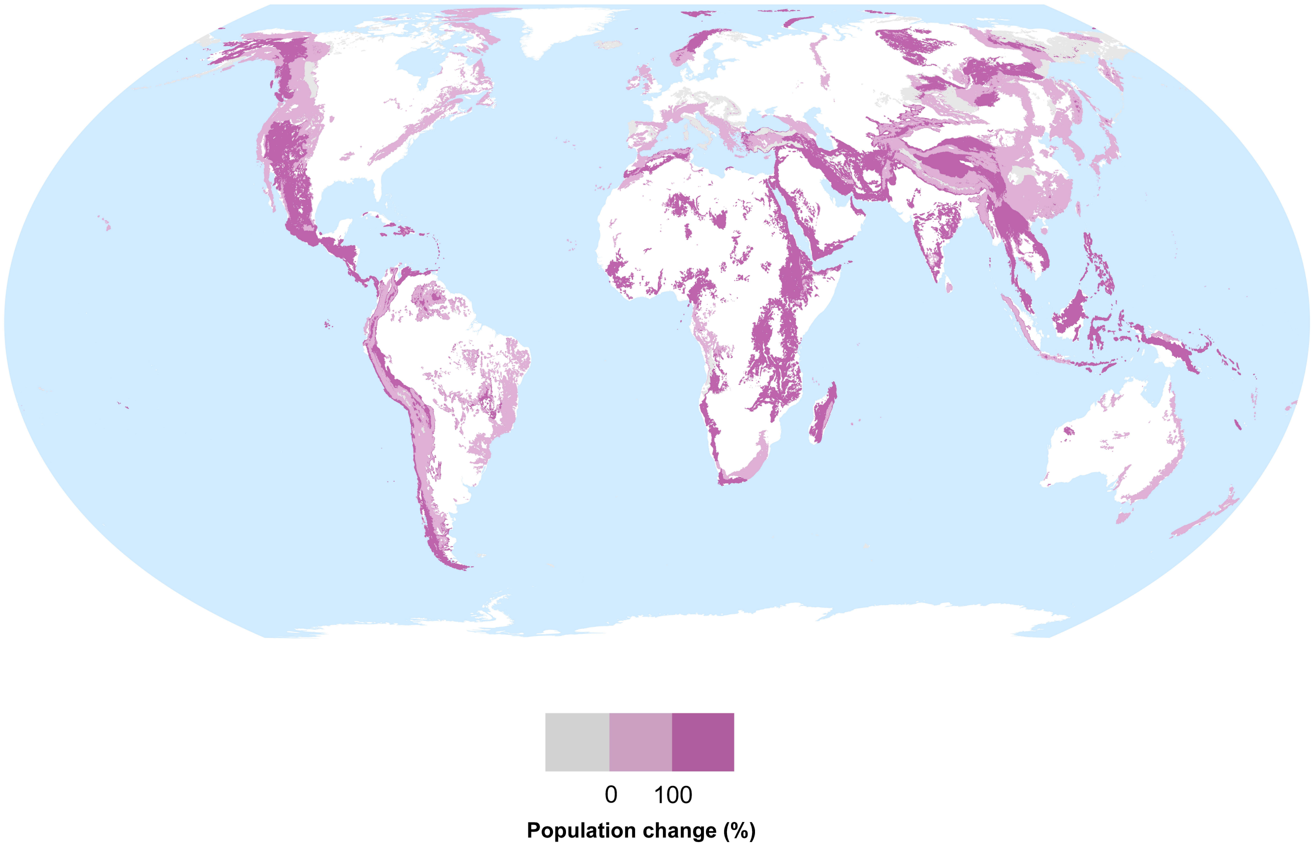

Fig. S11. Univariate choropleth map of population count change (%) between 1975 and 2015 by GMBA sub-mountain range zone according to the GHS-POP population dataset.

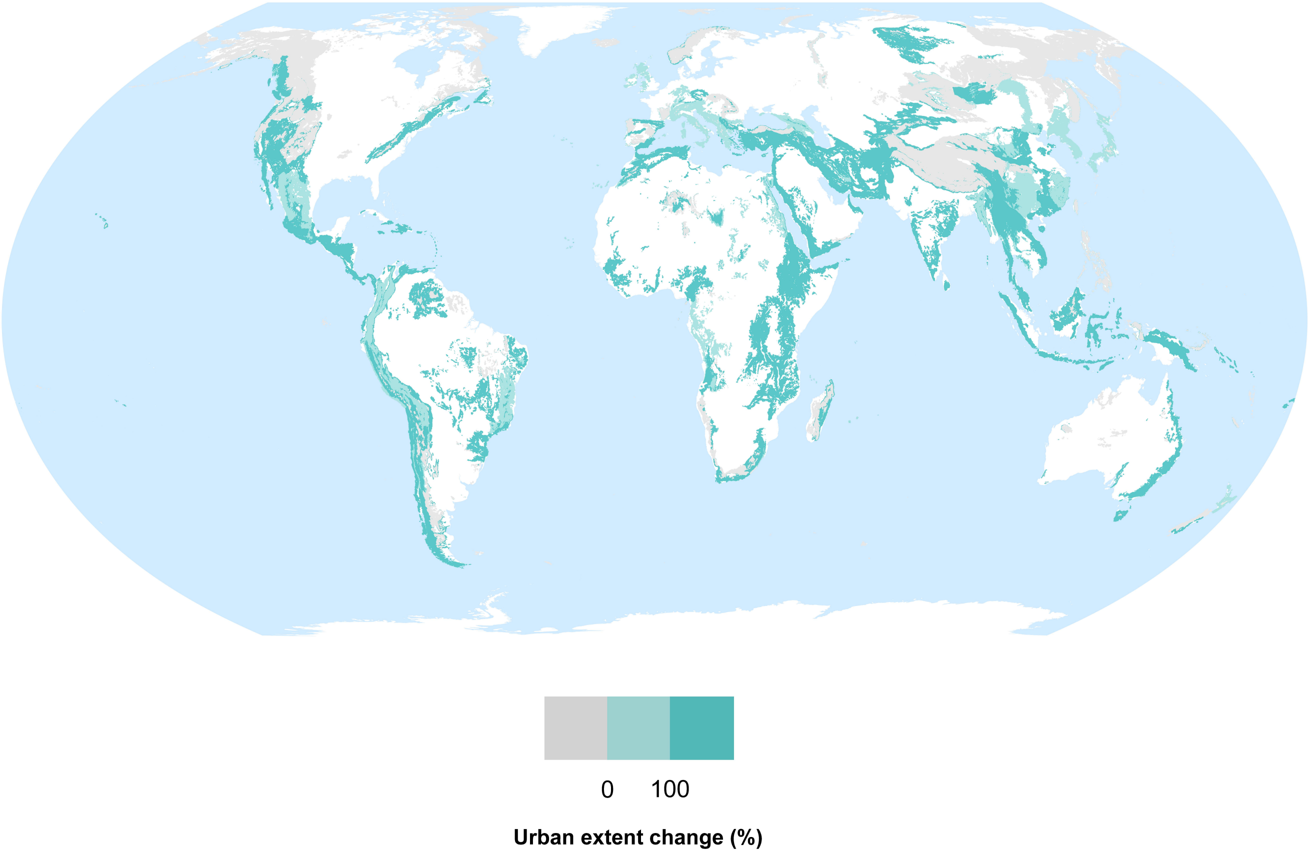

Fig. S12. Univariate choropleth map of urban extent change (%) between 1975 and 2015 by GMBA sub-mountain range zone according to the GHS-SMOD urban extent dataset.

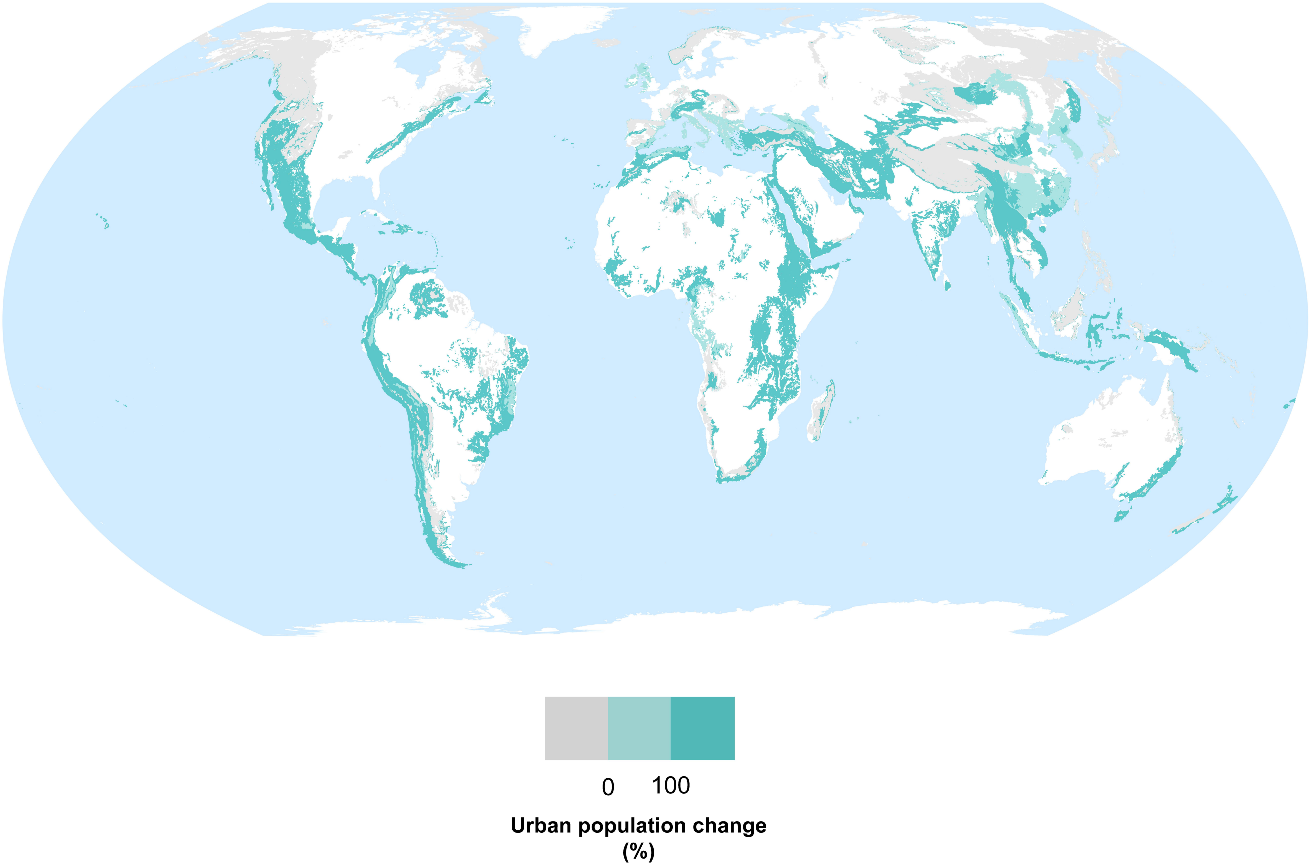

Fig. S13. Univariate choropleth map of urban population count change (%) between 1975 and 2015 by GMBA sub-mountain range zone according to the GHS-POP and GHS-SMOD datasets.

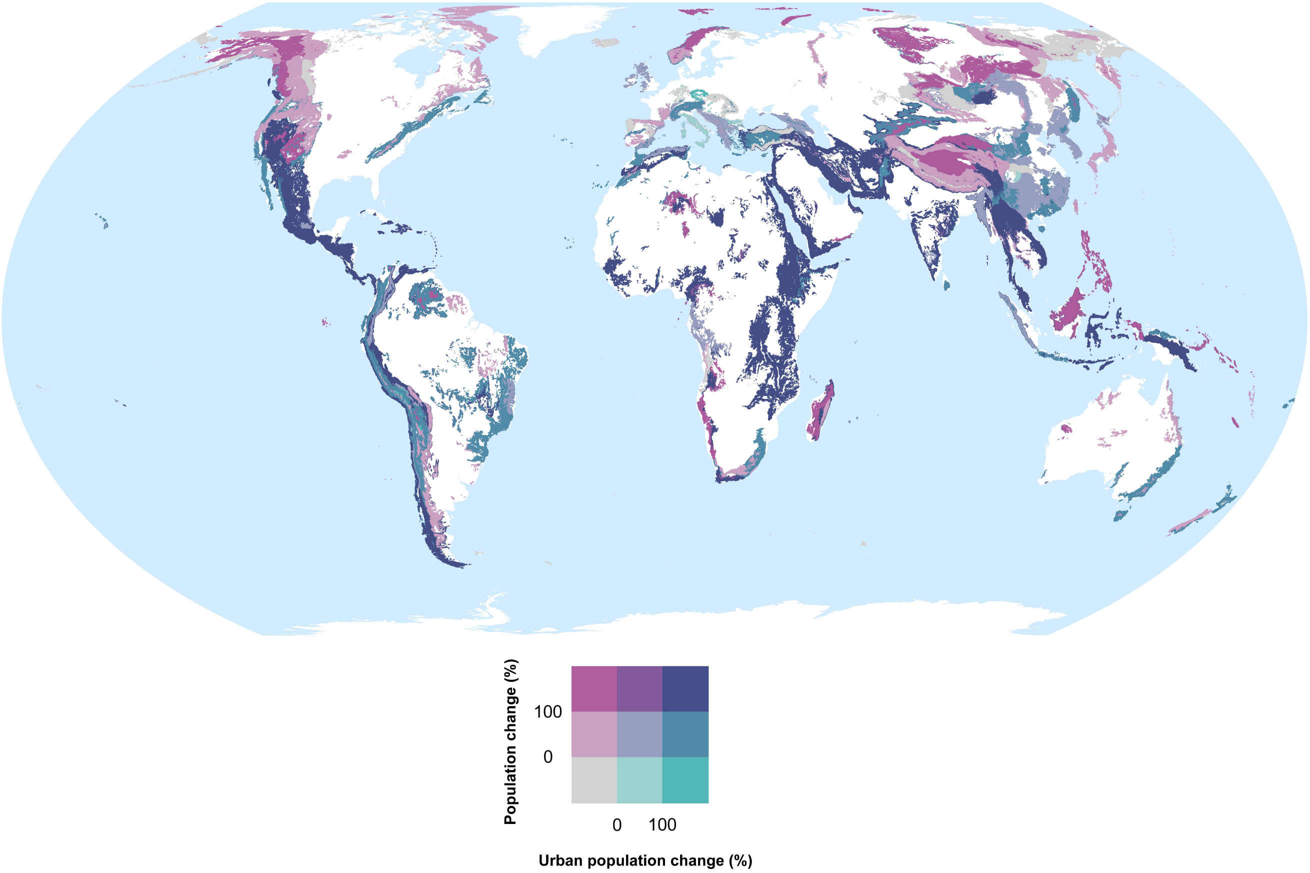

Fig. S14. Bivariate choropleth map of population count change vs. urban population count change (%) between 1975 and 2015 by GMBA sub-mountain range zone according to the GHS-POP and GHS-SMOD datasets

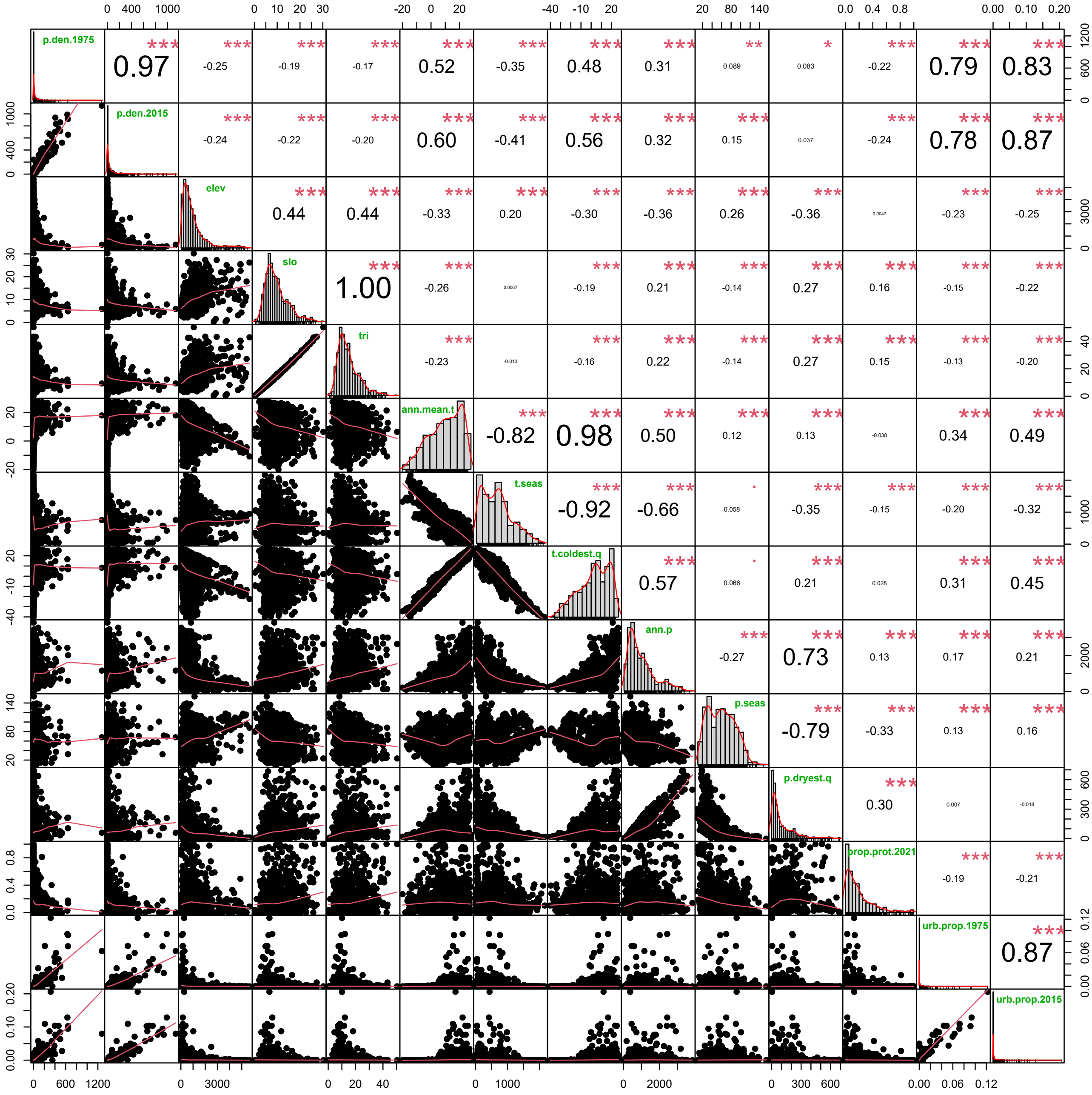

Fig. S15. More detailed version of the correlation matrix presented in Figure 8. This figure shows the univariate distribution of each variable (diagonal), bivariate scatter plots with fitted lines (lower), and correlation coefficients with an indication of their associated statistical significance denotes as follows: ***: 0 - 0.001, **: 0.001 - 0.01, *: 0.01 - 0.05, $\bullet$: $0.05 - 0.10$.

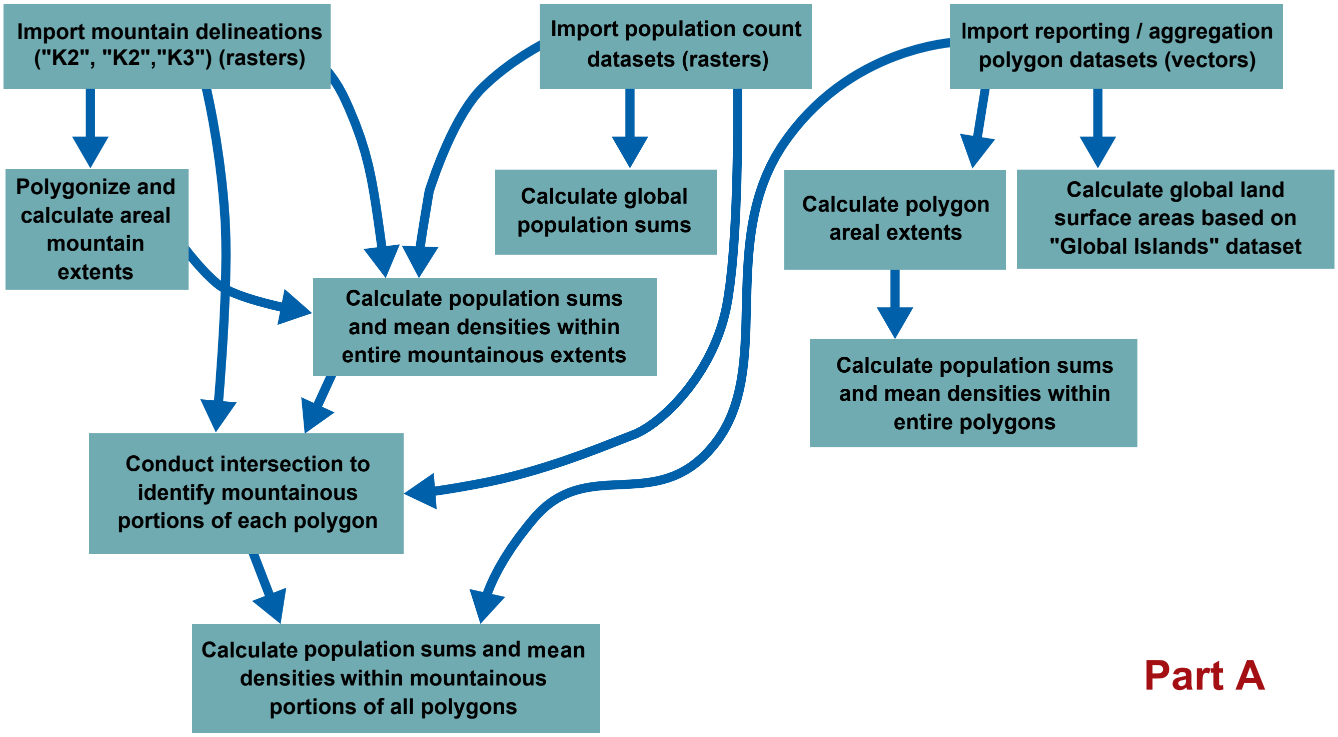

Fig. S16. Summary of the first phase ("Part A") of the workflow that was developed and applied. This phase was concerned with generating population count and mean density estimates at global level, at global level within the entire mountainous extent, within the entire reporting or aggregation polygon zones, and within only the mountainous portions thereof for, many mountain delineation/population dataset/reporting polygon combinations, and for as many years (or epochs) as appropriate.

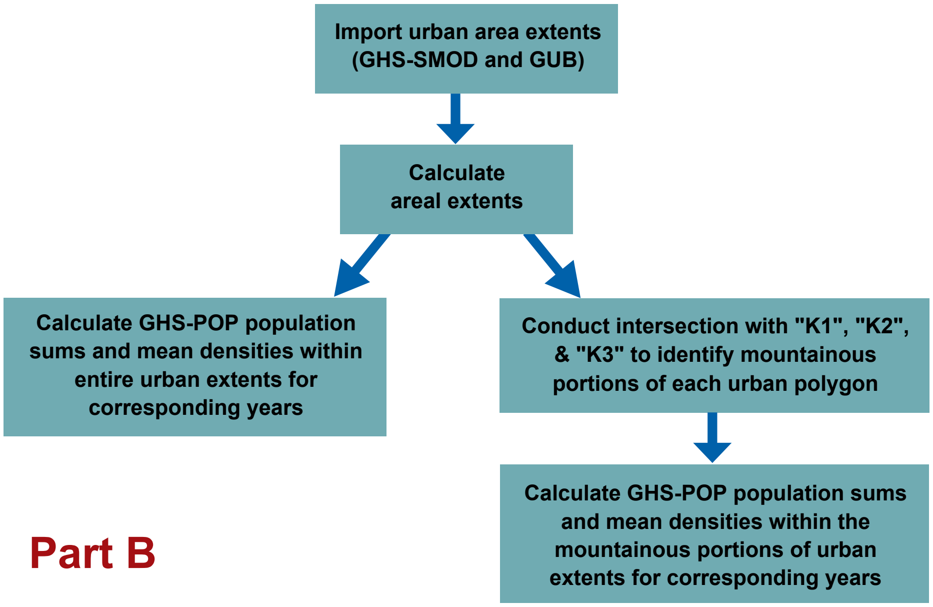

Fig. S17. Summary of the second phase ("Part B") of the workflow that was developed and applied. This phase was concerned with calculating urban extents and population metrics according to two alternative urban extent representations. For a single year (2015), the higher resolution WorldPop dataset was also used to give a point of comparison.

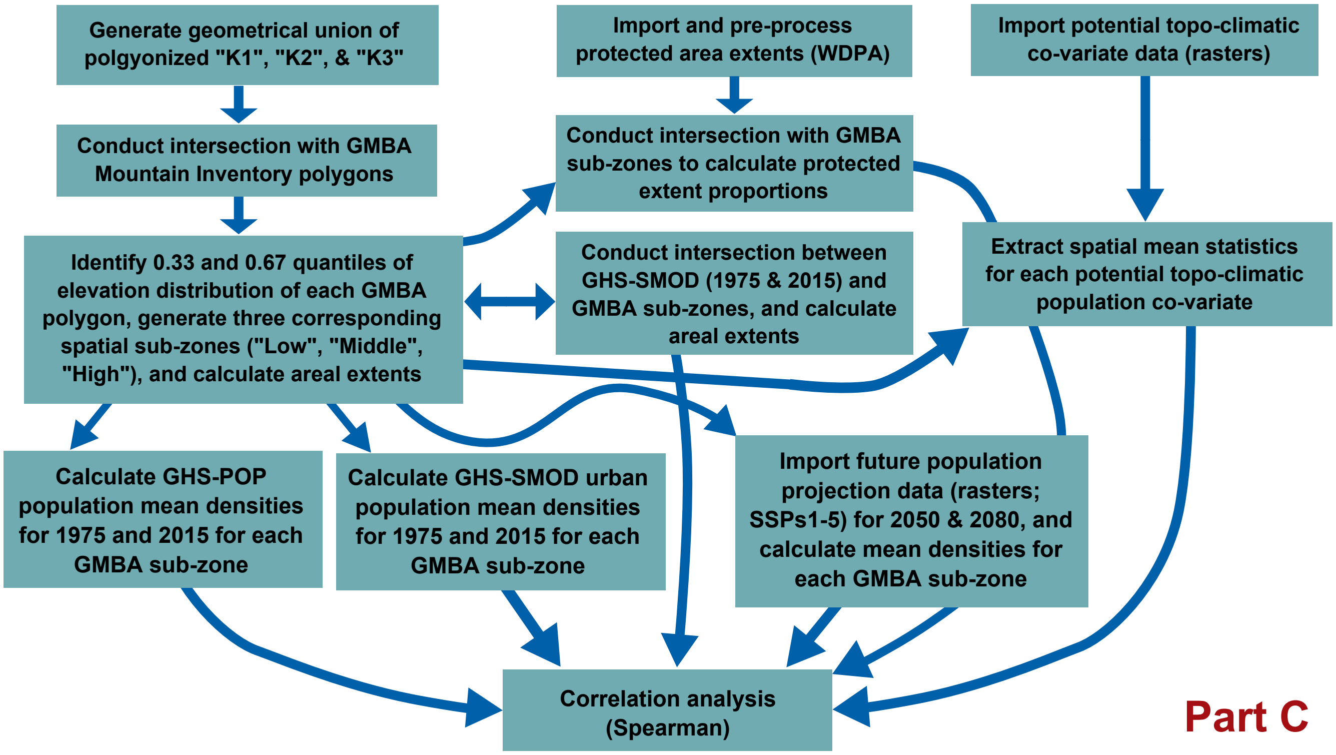

Fig. S18. Summary of the third phase ("Part C") of the workflow that was developed and applied. This phase was concerned with exploring the associations between population metrics and several potential co-variates at sub-mountain range scale (i.e. within individual mountain ranges). Future population estimates were extracted to assess the extent to which any historical dependencies are maintained.

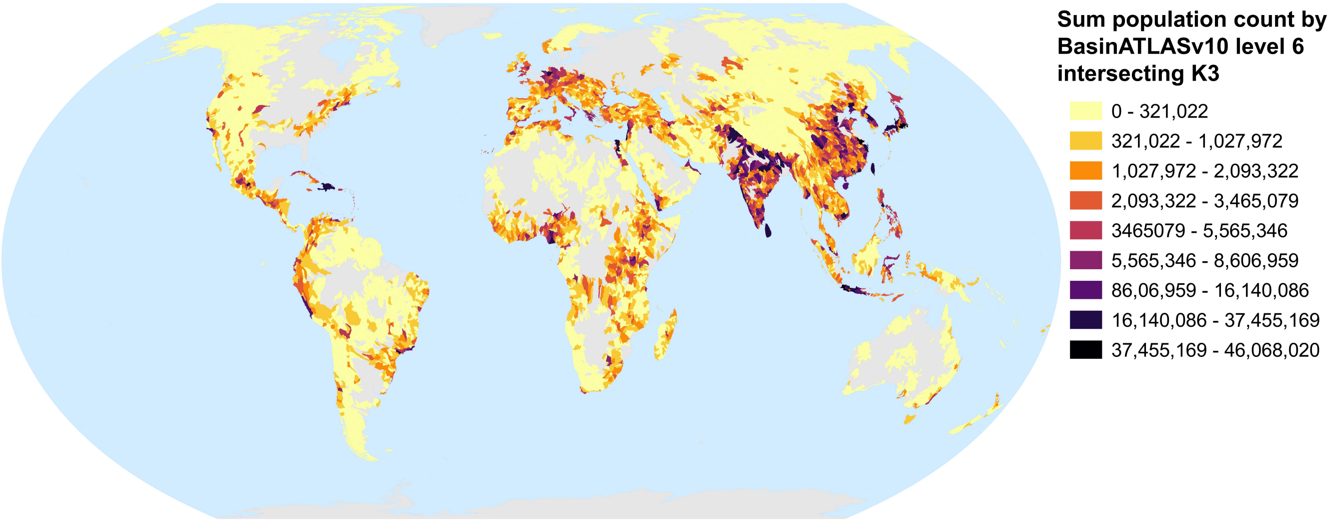

Fig. S19. Total population within the entire BasinATLASv10 level 6 catchment polygons that contain at least some mountainous terrain according to GPWv4 population dataset for 2015 and the K3 mountain delineation.

Table S1. Summary of the open-source mountain delineation datasets employed. All mountain delineation datasets were provided in a geographic projection system (WGS84; EPSG: 4326). Purely elevation-based mountain delineation criteria were not applied.

Name	Variable	Format	Spatial resolution (°)	Original Reference	Source
K1 (binary)	Mountain (Yes or No)	Raster (.tif)	0.00833	Kapos et al. (1)	Hyperlink
K2 (binary)	Mountain (Yes or No)	Raster (.tif)	0.00833	Körner et al. (2)	Hyperlink
K3 (binary)	Mountain (Yes or No)	Raster (.tif)	0.002083	Karagulle et al. (3)	Hyperlink

Table S2. Summary of the global gridded population count datasets employed. All population datasets were provided in a geographic projection system (WGS84; EPSG: 4326) *Past years are only considered for those datasets which support analyses of temporal changes. Data pertaining to 2015 are considered for all datasets to provide a comparison of the variability amongst the alternative population grids for a given year, however.

Name	Variable	Spatial resolution (°)	Reference(s)	Source	Year(s) considered*
Gridded Population of the World v4.11	Population count	0.00833	CIESIN (7); Doxsey-Whitfield et al. (8)	Hyperlink	2015
Global Human Settlement Layer GHS POP	Population count	0.0025	Florczyk et al. (9)	Hyperlink	1975,1990, 2000, 2015
LandScan	Population count	0.00833	Dobson et al. (10)	Hyperlink	2015, 2019
WorldPop	Population count	0.00083	Tatem (11)	Hyperlink	2015, 2020
Shared Socioeconomic Pathway (SSP) projections	Population count (total)	0.00833	Gao (12)	Hyperlink	2050, 2080

Table S3. Summary of the two data sources that were used to define urban extents.

Name	Attribute	Spatial resolution	Reference	Source	Years considered
GHS Settlement model grid (GHS-SMOD)	Code = 30	1 km (Mollweide equal area projection)	Pesaresi et al. (13)	Hyperlink	1975, 1990, 2000, 2015
Global Urban Boundaries (GUB)	N/A	N/A (but derived from 30 m data)	Li et al. (14)	Hyperlink	1990, 2000, 2015

Table S4. Summary of the aggregation / reporting polygon datasets employed. The number of zones refers to the number of distinct classes against which aggregated results are required, rather than the number of distinct feature polygons in the dataset overall (which depending on the layer can be considerably more.* To download the version of the GMBA Mountain Inventory used here, under "Custom Download" select "Extent: Broad" and "Layers: 300 selection".

Dataset	Unique ID Field(s)	Format	Number of zones	Reference	Source
IPCC WGII Regions	"Region"	Vector (.shp)	7	N/A	See online SI
GMBA Mountain Inventory v2 ("GMI_v2_beta_broad_210707_292")	"rangename"	Vector (.shp)	292	Snethlage et al. (4)	Hyperlink*
IPBES Regions and sub-regions	"area" and "Sub_region"	Vector (.shp)	17 (excluding Antarctica)	IPBES ICT documentation (https://ict.ipbes.net/)	Hyperlink
World Climate Regions	"gridcode"	Vector (.shp)	18	Sayre et al. (6)	See online SI
BasinATLAS v10 level 06	"ogc_fid"	Vector (.shp)	16,397	Linke et al. (5)	Hyperlink

Table S5. Summary of the potential co-variate data layers that were introduced to explore possible controls on mountain population metrics. *WDPA_WDOECM_May2021_Public.gdb

Data catalogue	Variable / feature	Spatial resolution (°)	Reference	Source	Year(s) considered
MERIT DEM	Elevation	0.000833	Yamazaki et al. (15)	Hyperlink	N/A
GMTED2010	Elevation	0.002083	Danielson and Gesch (16)	Hyperlink	N/A
Geomorpho90m	Slope (°)	0.000833	Amatulli et al. (17)	Hyperlink	N/A
Geomorpho90m	Terrain ruggedness index	0.000833	Amatulli et al. (17)	Hyperlink	N/A
WorldClim v2.1	Annual mean temperature (°C)	0.00833	Fick and Hijmans (18)	Hyperlink	Average 1970-2000
WorldClim v2.1	Temperature seasonality (standard deviation × 100)	0.00833	Fick and Hijmans (18)	Hyperlink	Average 1970-2000
WorldClim v2.1	Mean temperature of coldest quarter (°C)	0.00833	Fick and Hijmans (18)	Hyperlink	Average 1970-2000
WorldClim v2.1	Annual (sum) precipitation (mm)	0.00833	Fick and Hijmans (18)	Hyperlink	Average 1970-2000
WorldClim v2.1	Precipitation seasonality (Coefficient of Variation)	0.00833	Fick and Hijmans (18)	Hyperlink	Average 1970-2000
WorldClim v2.1	Precipitation of driest quarter (mm)	0.00833	Fick and Hijmans (18)	Hyperlink	Average 1970-2000
World Database on Protected Areas (WDPA)*	Protected area and “other effective area-based conservation measure” extents (version: May 2021)	N/A (polygon geometries)	UNEP-WCMC (2019)	Hyperlink	2021 (May)
Shared Socioeconomic Pathway (SSP) projections	Population count (total)	0.00833	Gao (12)	Hyperlink	2050, 2080

Table S6. Areal extents of the global mountain delineations that were employed in the present study. *The global land surface area (excluding Antarctica) was calculated according to the high-resolution global coastlines dataset of Sayre et al. (19), with "Very Small Islands" being excluded. All areas calculations for this paper were made on the spheroid. Note additionally that K1 considered a large part of the Greenland Ice Sheet to be mountainous. Mountainous regions in Antarctica and surrounding islands were removed from K1 prior to the analysis.

	**Total area (km^2)**	**Proportion of total global land surface**
**Global land surface area, excluding Antarctica***	135,073,414	1.00
K1	32,600,561	0.24
K2	17,336,688	0.13
K3	40,847,037	0.30

Table S7. Global and mountain-only population count data that were plotted to generate Figure 1.

Population dataset	Epoch	Population count [as proportion of corresponding global total]						
		Global total	Within K1		Within K2		Within K3	
GPW v4	2015	7,329,886,101	1,285,261,738	0.18	746,810,263	0.10	2,289,070,820	0.31
GHS POP	1975	4,061,342,585	559,333,406	0.14	214,793,759	0.05	1,187,381,039	0.29
GHS POP	1990	5,309,591,582	735,040,161	0.14	262,708,398	0.05	1,548,766,237	0.29
GHS POP	2000	6,126,524,095	850,580,100	0.14	293,988,129	0.05	1,774,477,441	0.29
GHS POP	2015	7,349,323,942	1,019,035,189	0.14	344,373,780	0.05	2,091,202,494	0.28
LandScan	2015	7,284,273,061	1,025,346,164	0.14	355,304,113	0.05	2,079,260,219	0.29
LandScan	2019	7,604,057,696	1,079,079,965	0.14	374,695,373	0.05	2,170,575,327	0.29
WorldPop	2015	7,969,633,182	1,098,625,475	0.14	498,110,749	0.06	2,150,490,122	0.27
WorldPop	2020	7,330,048,571	1,191,831,545	0.16	536,364,180	0.07	2,316,930,451	0.32

Table S8. Spearman's rank correlation coefficients between past and future projected population density, and potential explanatory variables. Red font denotes insignificant coefficients at the 0.05 confidence level.

	p.den.1975	p.den.2015	p.den.2050. ssp1	p.den.2050. ssp2	p.den.2050. ssp3	p.den.2050. ssp4	p.den.2050. ssp5	p.den.2080. ssp1	p.den.2080. ssp2	p.den.2080. ssp3	p.den.2080. ssp4	p.den.2080. ssp5
**ann.mean.t**	0.516	0.599	0.609	0.616	0.629	0.625	0.602	0.621	0.634	0.658	0.654	0.603
**ann.p**	0.309	0.318	0.374	0.373	0.372	0.375	0.375	0.375	0.372	0.366	0.376	0.376
**elev**	-0.249	-0.244	-0.205	-0.197	-0.184	-0.198	-0.211	-0.217	-0.204	-0.174	-0.203	-0.227
**p.dryest.q**	0.083	0.037	0.085	0.077	0.060	0.073	0.094	0.088	0.071	0.036	0.062	0.106
**p.seas**	0.089	0.149	0.127	0.140	0.165	0.145	0.114	0.117	0.141	0.192	0.150	0.089
**prop.prot.2021**	N/A	-0.240	-0.232	-0.236	-0.248	-0.232	-0.224	-0.216	-0.227	-0.254	-0.217	-0.197
**slo**	-0.189	-0.223	-0.099	-0.095	-0.093	-0.108	-0.098	-0.123	-0.121	-0.114	-0.142	-0.122
**t.coldest.q**	0.478	0.556	0.568	0.576	0.589	0.584	0.560	0.582	0.595	0.620	0.616	0.564
**t.seas**	-0.353	-0.412	-0.430	-0.438	-0.450	-0.446	-0.423	-0.445	-0.458	-0.480	-0.477	-0.429
**tri**	-0.167	-0.198	-0.074	-0.070	-0.068	-0.083	-0.074	-0.098	-0.096	-0.089	-0.117	-0.098
**urb.prop.1975**	0.792	N/A	N/A	N/A	N/A	N/A	N/A	N/A	N/A	N/A	N/A	N/A
**urb.prop.2015**	N/A	0.865	N/A	N/A	N/A	N/A	N/A	N/A	N/A	N/A	N/A	N/A

References

1. V Kapos, J Rhind, M Edwards, MF Price, C Ravilious, Developing a map of the world's mountain forests. *For. sustainable mountain development: a state knowledge report for 2000. Task Force on For. Sustain. Mountain Dev.* pp. 4–19 (2000).
2. C Körner, J Paulsen, EM Spehn, A definition of mountains and their bioclimatic belts for global comparisons of biodiversity data. *Alp. Bot.* **121**, 73–78 (2011).
3. D Karagulle, et al., Modeling global Hammond landform regions from 250-m elevation data. *Transactions GIS* **21**, 1040–1060 (2017).
4. MA Snethlage, et al., A hierarchical inventory of the world's mountains for global comparative mountain science. *Sci. Data* **9**, 149 (2022).
5. S Linke, et al., Global hydro-environmental sub-basin and river reach characteristics at high spatial resolution. *Sci. Data* **6**, 283 (2019).
6. R Sayre, et al., An assessment of the representation of ecosystems in global protected areas using new maps of World Climate Regions and World Ecosystems. *Glob. Ecol. Conserv.* **21**, e00860 (2020).
7. CIESIN, Gridded Population of the World, Version 4 (GPWv4): Population Count, Revision 11 (2018).
8. E Doxsey-Whitfield, et al., Taking Advantage of the Improved Availability of Census Data: A First Look at the Gridded Population of the World, Version 4. *Pap. Appl. Geogr.* **1**, 226–234 (2015).
9. A Florczyk, et al., GHSL Data Package 2019, (European Union), Technical report (2019).
10. JE Dobson, EA Bright, PR Coleman, RC Durfee, BA Worley, LandScan: a global population database for estimating populations at risk. *Photogramm. engineering remote sensing* **66**, 849–857 (2000).
11. AJ Tatem, WorldPop, open data for spatial demography. *Sci. Data* **4**, 170004 (2017).
12. J Gao, Global 1-km Downscaled Population Base Year and Projection Grids Based on the Shared Socioeconomic Pathways, Revision 01 (2020).
13. M Pesaresi, A Florczyk, M Schiavina, M Melchiorri, L Maffenini, GHS-SMOD R2019A - GHS settlement layers, updated and refined REGIO model 2014 in application to GHS-BUILT R2018A and GHS-POP R2019A, multitemporal (1975-1990-2000-2015), (European Commission, Joint Research Centre (JRC)), Technical report (2019).
14. X Li, et al., Mapping global urban boundaries from the global artificial impervious area (GAIA) data. *Environ. Res. Lett.* **15**, 094044 (2020).
15. D Yamazaki, et al., A high-accuracy map of global terrain elevations. *Geophys. Res. Lett.* **44**, 5844–5853 (2017).
16. J Danielson, D Gesch, Global Multi-resolution Terrain Elevation Data 2010 (GMTED2010). *U.S. Geol. Surv. Open-File Rep. 2011-1073* **2010**, 26 (2011).
17. G Amatulli, D McInerney, T Sethi, P Strobl, S Domisch, Geomorpho90m, empirical evaluation and accuracy assessment of global high-resolution geomorphometric layers. *Sci. Data* **7**, 162 (2020).
18. SE Fick, RJ Hijmans, WorldClim 2: new 1-km spatial resolution climate surfaces for global land areas. *Int. J. Climatol.* **37**, 4302–4315 (2017).
19. R Sayre, et al., A new 30 meter resolution global shoreline vector and associated global islands database for the development of standardized ecological coastal units. *J. Oper. Oceanogr.* **12**, S47–S56 (2019).
