# Supplementary material for: Human populations in the world’s mountains: Spatio-temporal patterns and potential controls
Source: PLoS One. 2022 Jul 20;17(7):e0271466. doi: 10.1371/journal.pone.0271466 (PMC9299344; doi:10.1371/journal.pone.0271466)
Supplement: Attachment — Submitted filename: Review_and_responses.pdf [file pone.0271466.s002.pdf]

ACADEMIC EDITOR: This is an excellent, well thought out study that presents interesting results and acknowledges, as well as quantifies the limitations of the data and methodologies used. The paper nearly meets Plos One criteria for publication. There is a large literature on the ecological/climate and social-economic correlates of human population density. The paper should better integrate with this literature and, I think, would be strengthened by acknowledging the correspondence of results across studies of global variation in human population density. My full review is appended below. I wish the authors the best with their revisions and interesting research.

Response: We are pleased that our work was well received. Whilst the analysis of potential population covariates is only part of our study (simply providing the comparative estimates being the other main part), during revision we have focused on integrating some of the existing literature on ecological/climate and social-economic correlates of human population density, as the Editor suggests, in order to further contextualise our results. The details of the modifications made are provided below. We hope our manuscript now fully meets journal's publication criteria.

Summary: This paper is an exploratory description and analysis of human settlement and density in mountainous regions. The authors quantify mountain population distributions using open source data and methodology. They also assess the robustness of their descriptions to changes in the data sets used, in particular data sets for estimating mountainous areas (K1,K2,and K3), as well as global estimates of human population. Finally, the authors identify covariates of mountain population density—like temperature. The authors demonstrate that the choice of data set, and the underlying methodology used to construct the data set, changes the results of their calculation of human population density and urban settlement. Further, the authors conclude that human population density in mountains correlates most strongly with temperature. The results help build a foundation for modeling the effects of climate change on the distribution of humans in mountains.

Substantive Comments:

(1) The authors could and should integrate their research better with previous work on the ecological and social-technological determinants of human population density. See references below. Although this work does not focus on mountains specifically, it does provide previous results relevant for predicting the factors that impact human population density in mountains. Given the close correspondence of the current paper's results and the results of these previous studies, **it seems worth noting that similar climate (ecological factors) associate with human population density, regardless of whether the data are restricted to mountains only**. Importantly, these non-linear relationships hold across different types of economic organization. This redundancy of results across different data sets and study methodologies may signal an important fundamental result useful for modeling human responses to climate change.

Response: Many thanks for this comment. We agree that some of the existing literature exploring global scale (or non-mountain specific) determinants of human population density chould have been better integrated into our paper. In our revised version, we have incorporated various additional citations and corresponding reflections, including the following:

- In the Introduction (see L119-123) of the revised version), we now reference the three additional papers (Luck, 2007; Tallavaara et al., 2018, Freeman et al. 2020) that analysed / review correlations between human population density and environmental factors on a global basis, in some cases for pre-industrialised populations. At this point, we emphasise that these analyses were not mountain specific and in some cases consider hunter-gather rather than contemporary societies. These changes serve to further emphasize novel aspects of our work (mountain specific, and dealing with population dynamics over recent decades).

- In the section where we report in our regional analysis that the population densities in many regions are lower for the mountainous regions than the entire regions within which they are bedded, we now mention that this is broadly consistent with the findings of Cohen and Small (1998), who found more people live at lower elevations than would be expected "by chance" (i.e. under an even spatial distribution), and that this is likely to due cooler temperatures and hence lower productivity at higher elevations (See L403 in the revised version).

- In the section where we report the negative association between human population densities in mountains and protected area extents, we mention that this finding is consistent with results of the large, global scale meta-analysis of Luck et al. (2007). (See L478 in the revised version)

- When discussing the possible implications of our findings around population correlations with temperature, we also cite Luck et al. (2007) in relation to the possible, even probable, migration of humans to cooler / more temperate regions; this was also identified as a likely future dynamic by that author, although not with specific reference to mountains (See L526 in the revised version).

- We have added a new section in our Outlook section stating that "Most previous (global) studies exploring correlations with human population employed more direct drivers, such as net primary productivity, biodiversity, and pathogen stress, as covariates, rather than the more indirect environmental conditions considered here", and that future mountain studies could seek to apply these more direct covariates explicitly." (L641 in the revised version).

- We have added a sentence to one of the bullet points in the conclusion rephrasing the fact that our findings are broadly coherent with the additional literature that was introduced during the revision (i.e. the "redundancy" the Editor mentions) (L744 in the revised version).

Minor Comments/reading Notes:

(1) Abstract should not exceed 300 words. I did not count the words, but it reads long. Please double check

Response: We checked this and found that the length of the abstract in the submitted version was only 272 words. Nevertheless, we have revised certain language in the abstract with the aim of increasing its flow and readability. The 300-word limit remains respected.

(2) "likewise responding to evolving conditions." Change `evolving' to 'changed' conditions

Response: We agree that "evolving" was not the best word choice in this context (potential for confusion, since the manuscript touches on topics related to biodiversity). That said, we prefer "changing" (rather than "changed") to give an indication of the ongoing nature of the changes in external climatic forcing, and have updated the revised manuscript accordingly. (L43 in the revised version).

(3) ``Elucidating any such relationships could lead to improved understanding of the underlying drivers, and could even enable the identification of topographic, climatic, and other conditions under which high population densities can develop and be sustained in mountains."

See references below on global investigations of the climate, ecology, and technological covariates of human population density: These should be reasonable places to start to understand the processes underlying variation in human population density. The results are relevant, though none focus on mountains specifically.

@article{Freemanetal2020,

title={The global ecology of human population density and interpreting changes in paleo-population density},

author={Freeman, Jacob and Robinson, Erick and Beckman, Noelle G and Bird, Darcy and Baggio, Jacopo A and Anderies, John M},

journal={Journal of Archaeological Science},

volume={120},

pages={105168},

year={2020},

publisher={Elsevier}

}

@article{Tallavaara2018,

title={Productivity, biodiversity, and pathogens influence the global hunter-gatherer population density},

author={Tallavaara, Miikka and Eronen, Jussi T and Luoto, Miska},

journal={Proceedings of the National Academy of Sciences},

volume={115},

pages={1232--1237},

year={2018},

publisher={National Acad Sciences}

}

@article{luck2007review,

title={A review of the relationships between human population density and biodiversity},

author={Luck, Gary W},

journal={Biological Reviews},

volume={82},

number={4},

pages={607--645},

year={2007},

publisher={Wiley Online Library}

}

Response: Many thanks for drawing our attention to these references, which we have read carefully and have incorporated material from during our revisions, as explained above. With respect to this

specific phrase, we have made a modification such that the text now reads "Elucidating the nature of such relationships in mountains..." (L128 in the revised version).

(4) ``these outputs are also provided are provided as vector files". Typo.

Response: Many thanks for spotting this error; it has now been fixed.

(5) ``However, in some more arid zones (Cool Temperate Dry, Subtropical Dry, and Tropical Desert), the situation is reversed, with mean mountain population densities higher."

I think that this observation fits with first principles of food availability and disease as important factors that impact human population density. In high latitude areas, mountains are cold and less productive. In dry temperate and dry tropical areas, mountains often receive more rainfall due to adiabatic cooling and provide an ``escape" from pathogen vectors.

Response: We have added an additional sentence (with citations) here to state that the finding that mountain population densities are generally highest in moist temperate conditions, which will be relatively high productivity, accords with previous global findings (L428 of the revised version).

With regards to mountain precipitation, we in fact mentioned this (that the generally higher precipitation totals may enable mountain regions to act as important refugia for people) in our original version (L379). In our revised version, we maintain this statement, and now link it to an earlier sentence in which we explicitly mention adiabatic cooling (L407) in the revised version.

(6) Important to note that the relationship between temperature and population density is non-linear. This may be due to an optimal thermal zone; however, the authors should see the papers cited above. At a global scale, net primary productivity and pathogen stress correlate with temperature. So, as productivity increases so does pathogen load. In theory, there should be a point where the costs of living at higher densities, in terms of disease transmission mortality and morbidity, outweigh the benefits of more productive ecosystems.

Response: Indeed, this is important. We have revised our text to mention explicitly that this relationship is non-linear (L507 in the revised version). In our original version, we did in fact mention that the fact that most population density increase have occurred in warm, but not extremely hot conditions (i.e. in an optimal thermal zone) (L459 in the original version). We also proposed such an optimal zone with respect to precipitation (L426 of the original version).

We have also added text regarding a possible interpretation of this bivariate plot involving productivity and pathogen stress (namely that since densities have increased in most warm zones, it may be that primary productivity has continued to outweigh pathogen stress until now) (L511 in the revised version). It is likely that the strength of this trade off in many mountain regions (as elsewhere) will be modulated not only by the extent to which modern agricultural technologies are deployed, but perhaps even more importantly by the nature of the physical infrastructure, sanitation, and healthcare systems (which were not considered here). As such, in more developed / industrialised societies, it may be that human thermal "comfort", or the amount of annual cooling or heating needed, as well as water resources (influenced by precipitation) are more important drivers.

Please submit your revised manuscript by Jul 03 2022 11:59PM. If you will need more time than this to complete your revisions, please reply to this message or contact the journal office at plosone@plos.org. When you're ready to submit your revision, log on to https://www.editorialmanager.com/pone/ and select the 'Submissions Needing Revision' folder to locate your manuscript file.

Please include the following items when submitting your revised manuscript:

• A rebuttal letter that responds to each point raised by the academic editor and reviewer(s). You should upload this letter as a separate file labeled 'Response to Reviewers'.

• A marked-up copy of your manuscript that highlights changes made to the original version. You should upload this as a separate file labeled 'Revised Manuscript with Track Changes'.

• An unmarked version of your revised paper without tracked changes. You should upload this as a separate file labeled 'Manuscript'.

If you would like to make changes to your financial disclosure, please include your updated statement in your cover letter. Guidelines for resubmitting your figure files are available below the reviewer comments at the end of this letter.

We look forward to receiving your revised manuscript.

Kind regards,

Jacob Freeman

Academic Editor

PLOS ONE

Journal Requirements:

When submitting your revision, we need you to address these additional requirements.

1. Please ensure that your manuscript meets PLOS ONE's style requirements, including those for file naming. The PLOS ONE style templates can be found at https://journals.plos.org/plosone/s/file?id=wjVg/PLOSOne_formatting_sample_main_body.pdf and

https://journals.plos.org/plosone/s/file?id=ba62/PLOSOne_formatting_sample_title_authors_affiliations.pdf

2. Thank you for stating the following in the Acknowledgments Section of your manuscript:

"We are extremely grateful to the developers and maintainers of the numerous open datasets and open-source software packages used. J.M.T, C.A., and P.W. acknowledge funding from the Swiss Agency for Development and Cooperation (SDC) (Project number: 7F-10208.01.02). We thank Mr. Matthias Fries for IT support, Prof. C. Körner for useful discussions, and Dr. E. Beever for thoughtful comments on an earlier draft."

We note that you have provided funding information that is not currently declared in your Funding Statement. However, funding information should not appear in the Acknowledgments section or other areas of your manuscript. We will only publish funding information present in the Funding Statement section of the online submission form.

Please remove any funding-related text from the manuscript and let us know how you would like to update your Funding Statement. Currently, your Funding Statement reads as follows:

"J.M.T, C.A., and P.W. acknowledge funding from the Swiss Agency for Development and Cooperation (SDC; https://www.eda.admin.ch/sdc) (Project number: 7F-10208.01.02).

GI acknowledges funding from the Institute of Geography, Russian Academy of Sciences (http://www.igras.ru/en/node/1) (Project number: 0148-2019-0007)

The sponsors played no direct role in the study design, data collection and analysis, decision to publish, or preparation of the manuscript."

Please include your amended statements within your cover letter; we will change the online submission form on your behalf

Response: As requested, we have removed all funding information from the revised manuscript itself. Our amended funding statement is provided above; many thanks for changing the online submission form on our behalf:

Please review your reference list to ensure that it is complete and correct. If you have cited papers that have been retracted, please include the rationale for doing so in the manuscript text, or remove these references and replace them with relevant current references. Any changes to the reference list should be mentioned in the rebuttal letter that accompanies your revised manuscript. If you need to cite a retracted article, indicate the article's retracted status in the References list and also include a citation and full reference for the retraction notice.

Response: In the revised version, we have included the additional references recommended by the Academic Editor (please see above). We have also updated / added references for the publication and full dataset citation corresponding to one of the key input datasets used in this study – the GMBA Mountain Inventory – which came out earlier in 2022 (Snethlage et al., 2022a,b). We furthermore now make reference to the IPCC Cross Chapter Paper on Mountains (Adler et al., 2022a,b), to which this work contributed, as well as a few other papers. The new references are marked in our "tracked changes" version. We confirm that we cite no articles that have since been retracted.

[Note: HTML markup is below. Please do not edit.]

Reviewers' comments:

Reviewer's Responses to Questions

Comments to the Author

1. Is the manuscript technically sound, and do the data support the conclusions?

The manuscript must describe a technically sound piece of scientific research with data that supports the conclusions. Experiments must have been conducted rigorously, with appropriate controls, replication, and sample sizes. The conclusions must be drawn appropriately based on the data presented.

Reviewer #1: Yes

---

2. Has the statistical analysis been performed appropriately and rigorously?

Reviewer #1: Yes

---

3. Have the authors made all data underlying the findings in their manuscript fully available?

The PLOS Data policy requires authors to make all data underlying the findings described in their manuscript fully available without restriction, with rare exception (please refer to the Data Availability Statement in the manuscript PDF file). The data should be provided as part of the manuscript or its supporting information, or deposited to a public repository. For example, in addition

to summary statistics, the data points behind means, medians and variance measures should be available. If there are restrictions on publicly sharing data—e.g. participant privacy or use of data from a third party—those must be specified.

Reviewer #1: Yes

---

4. Is the manuscript presented in an intelligible fashion and written in standard English?

PLOS ONE does not copyedit accepted manuscripts, so the language in submitted articles must be clear, correct, and unambiguous. Any typographical or grammatical errors should be corrected at revision, so please note any specific errors here.

Reviewer #1: Yes

Response: We are pleased that Reviewer#1 seems to have a positive impression of our manuscript.

---

5. Review Comments to the Author

Please use the space provided to explain your answers to the questions above. You may also include additional comments for the author, including concerns about dual publication, research ethics, or publication ethics. (Please upload your review as an attachment if it exceeds 20,000 characters)

Reviewer #1: It is advisable

1. to specify abbreviations immediately after the first use of the term.

Response: Indeed, in the original version, IPCC, IPBES, and WCR were not specified upon their first use. Many thanks for pointing this out. We have now made the necessary correction (see e.g. L209-210, 220). We have also carefully re-read the manuscript to ensure that all other abbreviations are specified upon first use.

2. Revise and simplify the Materials and Methods

Response: As requested, we have sought to revise, simplify, and slightly reorganise the Materials and Methods section in the revised version. It is always somewhat tricky to fully and concisely describe a complex algorithm or workflow in prose form, however, and for this reason the code itself (which is commented) should be referred to in case of any doubt.

3. Check and correct the confusing lines eg. it becomes confusing While explaining K1, K2, and K3 and jumping directly to class 7 of K1 in last line of para 3 under Materials and Methods.

Response: We have modified the text describing our decision and associated pre-processing in relation to class 7 of K1, and are confident that this is now clearer (L180 in the revised manuscript).

---

6. PLOS authors have the option to publish the peer review history of their article (what does this mean?). If published, this will include your full peer review and any attached files.

If you choose "no", your identity will remain anonymous but your review may still be made public.

Do you want your identity to be public for this peer review? For information about this choice, including consent withdrawal, please see our Privacy Policy.

Reviewer #1: Yes: Dr MD. ASIF IQUBAL

Other changes: We have slightly modified the format of Figure 1 to minimise the issue of some of the points obscuring one another. In addition, we have carefully read the manuscript have corrected a few small additional typographical errors, repositioned certain sections within the manuscript, and made certain other edits to improve readability / flow.

While revising your submission, please upload your figure files to the Preflight Analysis and Conversion Engine (PACE) digital diagnostic tool, https://pacev2.apexcovantage.com/. PACE helps ensure that figures meet PLOS requirements. To use PACE, you must first register as a user. Registration is free. Then, login and navigate to the UPLOAD tab, where you will find detailed instructions on how to use the tool. If you encounter any issues or have any questions when using PACE, please email PLOS at figures@plos.org. Please note that Supporting Information files do not need this step.
